# Computed Tomography Assessment of the Retrolabyrinthine Approach

**DOI:** 10.7759/cureus.38394

**Published:** 2023-05-01

**Authors:** Francisco Vaz-Guimaraes, Henrique Q Cartaxo, João E da Fonte, Marcelo M Valença

**Affiliations:** 1 Neurological Surgery, Real Hospital Português, Recife, BRA; 2 Radiology and Diagnostic Imaging, Real Hospital Português, Recife, BRA; 3 Neurological Surgery, Federal University of Pernambuco, Recife, BRA

**Keywords:** pneumatization of the mastoid, jugular bulb, sigmoid sinus, computed tomography, retrolabyrinthine approach

## Abstract

Introduction

This study aimed to evaluate preoperative radiological assessments of the retrolabyrinthine approach to identify and describe anatomical constraints that may anticipate a more challenging situation for neurosurgeons and otolaryngologists specialized in skull base surgery.

Materials and methods

The study included 75 adult patients who underwent high-resolution computed tomography angiography scans of the head, with the aim of analyzing the side of the dominance of the sigmoid sinus (SS), the level of pneumatization of the mastoid portion of the temporal bone, and the height of the jugular bulb.

Results

The results showed that dominant SS and type 2 jugular bulbs were more common on the right side, while smaller type 1 bulbs were significantly more common on the left.

Conclusions

These findings provide valuable information for neurosurgeons and otolaryngologists in predicting the difficulty of the retrolabyrinthine approach based on preoperative radiological assessments.

## Introduction

The choice of a surgical approach to treat intracranial diseases necessitates a comprehensive understanding of the brain and cranial base anatomy [1]. The retrolabyrinthine and the retrosigmoid approaches are commonly used to treat tumors located in the posterior cranial fossa such as vestibular schwannomas. It consists in the removal of portions of the petrous segment of the temporal bone (i.e. posterior petrosectomy) with preservation of the bony labyrinth and portions of the squamous portion of the occipital bone, respectively [2,3]. The decision to pursue one instead of another is predicted, among other factors, on patient anatomy [1,4].

The retrolabyrinthine approach was considered “the unsung hero” of skull base surgery. It provides shorter working distance and less cerebellar retraction than the traditional retrosigmoid approach [5]. The Trautmann’s triangle, an area of dura delimited by the superior petrosal sinus, the posterior semicircular canal (SCC), the jugular bulb and the sigmoid sinus (SS), provides surgical access to the posterior fossa through the retrolabyrinthine approach. However, the ease of the approach may be significantly affected due to significant variability in bony anatomy.

Therefore, this manuscript aims to evaluate preoperative radiological assessments of the retrolabyrinthine approach to identify and describe anatomical constraints that may anticipate a more challenging situation for neurosurgeons and otolaryngologists specialized in skull base surgery.

## Materials and methods

With the approval of the Institutional Review Board at Real Hospital Português (approval number 6.026.694), we retrospectively reviewed 75 consecutive adult patients of both genders, who underwent high-resolution computed tomography angiography (CTA) scans of the head between January 2020 and January 2022 at Real Hospital Português, a single large tertiary care and teaching institution. These scans were obtained in the axial plane with a 128-row CT scanner (Somatom Definition AS, Siemens, Erlangen, Germany) and a 0.8mm slice thickness. Reformatted images were then obtained from a volumetric isotropic dataset by using a dedicated workstation (Philips, Best, Netherlands) [6]. Standard axial, coronal and sagittal images were reconstructed at 0.1mm increments. Afterwards, images were electronically transferred to the Brainlab Elements software (Brainlab AG, Feldkirchen, Germany) for measurements, which was done by using its digital ruler and goniometer.

Patients less than 18 years old of age with current or past medical history of cranial surgery, cranial malformations, cranial trauma, and otological diseases were excluded. We analyzed the images for three main variables as follows: (A) The side of dominance of the SS, (B) the level of pneumatization of the mastoid portion of the temporal bone and (C) the height of the jugular bulb. We categorized the measurements using predetermined criteria previously published elsewhere [7-9]. We used the measurement ratio right/left for SS dominance. A sinus was classified as dominant when the measurement was > 1.5 (right-sided dominance) or < 0.67 (left-sided dominance), and co-dominant when the ratio was ≥ 0,67 and ≤ 1.5 [7]; The pneumatization of the mastoid cells was measured in relation to the SS and labyrinth on axial CTA imaging. It was classified as type 1 when pneumatization stopped anterior to the SS, type 2 when it extended halfway around the SS, type 3 when it extended to the posterior limit of the SS, and type 4 when it extended beyond this posterior limit [8]; the height of the jugular bulb was based on its location. It was classified as type 1 when there was no bulb, type 2 when located below the inferior margin of the posterior SCC, type 3 when located between the inferior margin of the posterior SCC and inferior margin of the internal acoustic canal, and type 4 when located above the inferior margin of the internal acoustic canal [9].

The categorical variables were described as frequencies and percentages and Fisher exact test was used for statistical comparison. Data were collected using Microsoft Excel 2020 (Microsoft Corp.). Analysis were performed with SPSS 21.0 software package (IBM Corp., Armonk, NY). All tests were 2-sided at alpha = 0.05.

## Results

The study population consisted of 35 male and 40 female patients with a mean age of 46,6 ± 17,1 years. A dominant SS was identified in 51 patients (68%), 36 on the right (48%) and 15 on left (20%), while a co-dominant SS was identified in 24 patients (32%). Females had a significantly higher incidence of left-sided dominance of the SS than males (30% versus 8.6%, p=0.024), although a dominant SS on the right was more common on both groups (54,29% and 42,5%, respectively) (Table 1).

**Table 1:** Dominance of the SS, gender and laterality Legend: Masc.: masculine; Fem.: feminine; Dom.: Dominance; *statistically significant

Gender	Type of SS Dominance			
	Right-sided Dom.	Co-Dom.	Left-sided Dom.	Total
Masc.	19	13	3	35
Fem.	17	11	12*	40
Total	36 (48%)	24 (32%)	15 (20%)	75

In terms of pneumatization level, we found type 1 in 11 mastoids (7,33%), type 2 in 51 (34%), type 3 in 28 (18.67%) and type 4 in 60 (40%). Type 2 was significantly more common on the right (33/75) than on the left (18/75) (p=0.009), while type 4 was significantly more common on the left (37/75) than on the right (23/75) (p=0.029) (Table 2).

**Table 2:** Pneumatization of mastoid cells, gender and laterality Legend: Masc.: masculine; Fem.: feminine; *statistically significant

Side Gender	Pneumatization of Mastoid Cells Type				
	1	2	3	4	Total
Right side	5	33 (44%)*	14	23 (30,67%)	75
Masc./Fem.	1/4	19/14	6/8	9/14	
Left side	6	18 (24%)	14	37 (49,33%)*	75
Masc./Fem.	5/1	5/13	6/8	19/18	
Total	11 (7,33%)	51 (34%)	28 (18,67%)	60 (40%)	150

The height of the jugular bulb was classified as type 1 in 11 patients (7.33%), type 2 in 89 (59.33%), type 3 in 40 (26.67%), and type 4 in 10 (6.67%). Type 1 bulbs were significantly more common on the left (10/75) than on the right (1/75) (p=0.009), while type 2 were significantly more common on the right (51/75) than on the left (38/75) (p=0.046). High-riding jugular bulbs (types 3 and 4) were more common on the left (27/75) than on the right (23/75) but this difference was not significant (p=0.603) (Table 3).

**Table 3:** Type of jugular bulb Legend: Masc.: masculine; Fem.: feminine; *statistically significant

Side Gender	Jugular Bulb Type				
	1	2	3	4	Total
Right side	1 (1,33%)	51 (68%)*	17	6	75
Masc./Fem.	1/0	24/27	7/10	3/3	
Left side	10 (13,33%)*	38 (50,67%)	23	4	75
Masc./ Fem.	7/3	20/18	7/16	1/3	
Total	11 (7,33%)	89 (59,33%)	40 (26,67%)	10 (6,67%)	150

## Discussion

**Figure 1:**
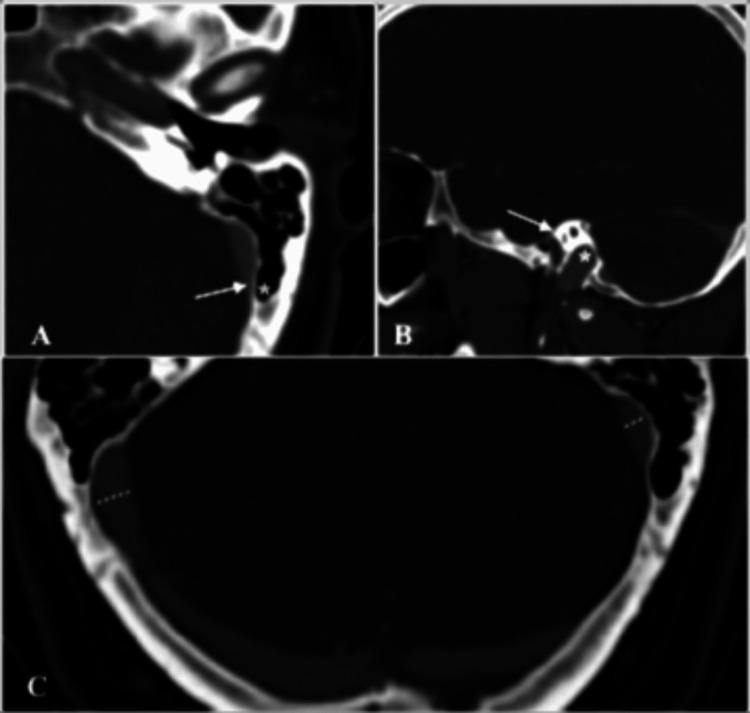
Computed tomography angiographies of the head A: axial view of the left temporal bone demonstrates a type 4 level of pneumatization of the mastoid with an aerated mastoid cell (asterisks) extending beyond the posterior limit of the sigmoid sinus (arrow); B: sagittal view of the right temporal bone demonstrates a type 3 jugular bulb with its dome (asterisks) located above the inferior margin of the posterior SCC (arrow); C: axial view of the posterior fossa demonstrates a right-side dominant SS (dotted lines ratio > 1.5), a type 2 pneumatization on the right and type 4 pneumatization on the left.

In this study we investigated the prevalence and laterality of various types of jugular bulbs and sigmoid sinuses (SS) as well as the level of pneumatization of the mastoid in a cohort of patients who underwent computed tomography angiography (CTA) imaging of the head. We found that dominant SS and type 2 jugular bulbs were more common on the right side, while smaller type 1 bulbs were significantly more common on the left side. Dominant SS on the left were also more common in female patients. It was also observed that the level of pneumatization was significantly higher on the left compared to the right side (Figure 1).

Other studies have reported that SS were right-side dominant or co-dominant [10]. Another radiographic study [11] has also observed a predominant right dominance of the SS (74% of 38 temporal bones) while other clinical studies have reported 60% of right-side dominance [12]. On the other hand, two angiographic studies [7,13] have observed a right-sided dominance between 29.3% and 35%, while co-dominance have been the most common pattern and observed between 49% and 50% of the patients. In terms of the height of the jugular bulb, one study reported a high jugular bulb was observed in 9.5% of 4598 temporal bones [14]. Lastly, in terms of the level of pneumatization of the mastoids, one study reported a normally distributed degree of pneumatization with no correlation with gender and laterality [15], while another study observed a higher degree of pneumatization (types 3 and 4) in their entire cohort of 20 temporal bones [16]. Compared to our study, these differences may be attributed to ethnical difference in the population or, most likely, differences in measurement techniques.

Previous studies have shown that larger and more prominent SS and jugular bulbs can limit the working space for a retrolabyrinthine approach to the posterior fossa, thus increasing the difficulty and risk of surgery. Therefore, accurate preoperative evaluation of these structures is crucial to ensure safe and effective surgery. Cho and Al-Mefty have suggested combining multiple surgical approaches to overcome these anatomical constraints during petroclival meningioma resection [17]. In addition, limited access to the internal auditory canal (IAC) has been reported in the presence of anteriorly displaced and likely larger and dominant SS and higher jugular bulbs [18-20].

The level of pneumatization of the mastoid is also an important factor in the retrolabyrinthine approach. Significant pneumatization may facilitate the identification of important anatomical landmarks such as the labyrinth and the SS, while poor pneumatization may increase the risk of inadvertent injury and make the exposure technically more challenging and time-consuming [4,18]. We observed that increasing levels of pneumatization were more commonly found on the contralateral side of the dominant SS, and hypothesized that it may be possibly due to a larger venous apparatus promoting increased osteoblastic activity and decreased pneumatization.

This study provides important descriptive data in regard to the CT assessment of the retrolabyrinthine approach. Nonetheless, it presents some limitations. The lack of anatomical and clinical correlations reduces its practical applicability. Other factors such as the area of exposure of Trautmann's triangle, the position of the SS, the petroclival angle, clival depth, and angle of attack to the IAC may also influence preoperative planning. Future studies are needed to investigate these factors further.

## Conclusions

The preoperative radiological assessment of the retrolabyrinthine approach is a step of utmost importance for neurosurgeons and otolaryngologists specialized in skull base surgery. The presence of dominant SS, higher jugular bulbs, and decreased level of pneumatization of the mastoids on the right side may indicate a more challenging surgical situation. Therefore, careful evaluation of these anatomical constraints is recommended for surgeons performing this type of operation.
